# Real-world effectiveness, safety, and health-related quality of life in people living with HIV receiving bictegravir/emtricitabine/tenofovir alafenamide—12-month results of the BICSTaR French cohort

**DOI:** 10.1016/j.ijregi.2025.100685

**Published:** 2025-06-17

**Authors:** Laurent Hocqueloux, Fabrice Bonnet, Claudine Duvivier, Matteo Vassallo, Franck Tollinchi, Pascale Leclercq, Marina Karmochkine, Hugues Cordel, Colin Deschanvres, Alicia C. Gordon, David Thorpe, Tali Cassidy, Francois Durand, Sabrinel Sahali, Olivier Robineau

**Affiliations:** 1 CHU d’Orléans, Orléans, France; 2 CHU de Bordeaux, and Université de Bordeaux, INSERM U 1219, Bordeaux Population Health Center, Bordeaux, France; 3 Paris Cité University, APHP Necker hospital; IHU Imagine; Institut Pasteur, Centre d’infectiologie Necker-Pasteur, INSERM U1016, CNRS UMR8104, Institut Cochin, Paris, France; 4 CH de Cannes, Cannes, France; 5 Hôpital Saint Joseph, APHM, Marseille, France; 6 CHU de Grenoble, Grenoble, France; 7 Hôpital Hôtel Dieu Paris, APHP, Paris, France; 8 Hôpital Avicenne, APHP, Paris, France; 9 Hôpital Hotel Dieu, Nantes, France; 10 Internal Medicine and Immunology department, Hôpital Bicêtre, Service de Médecin Interne et Immunologie Clinique, APHP, Paris, France; 11 Gilead Sciences, Uxbridge, UK; 12 Gilead Sciences, Boulogne-Billancourt, France; 13 EA2694, Univ Lille, Centre Hospitalier de Tourcoing, Tourcoing, France

**Keywords:** HIV infection, Antiretroviral therapy, Bictegravir, Tenofovir alafenamide, Real-world data

## Abstract

**Objectives:**

BICSTaR is a multinational, prospective, observational study that aimed to evaluate bictegravir/emtricitabine/tenofovir alafenamide (B/F/TAF) in HIV treatment-naïve (TN) and treatment-experienced (TE) participants in routine clinical practice.

**Methods:**

Month 12 analysis of the French cohort with respect to virologic effectiveness, drug-related adverse events (DRAEs), emergence of resistance, body weight, and patient-reported outcomes using the HIV Symptom Index and the HIV Treatment Satisfaction Questionnaire.

**Results:**

A total of 240 participants initiated B/F/TAF in January-July 2019 (56 TN, 184 TE), 79% of whom were male, with a median age of 50 years. At baseline, 63% (TN: 46%, TE: 68%) presented with comorbidities. At month 12, HIV-1 RNA was <50 cp/mL in 92% (43/47) of TN and 96% (134 of 139) of TE in missing = excluded analysis (discontinuation = failure analysis: TN: 92% [43 of 47], TE: 92% [134 of 146]). No major mutations associated with B/F/TAF resistance emerged. A total of 7% (16 of 240) discontinued B/F/TAF, including 4% (10 of 240) due to DRAEs and none for virologic reasons. DRAEs were reported in 13% (30 of 240) (no renal DRAE). The median changes in body weight were +6.5 kg in TN and +1.0 kg in TE. The number of bothersome symptoms decreased in the TN group, and treatment satisfaction significantly increased in the TE group.

**Conclusions:**

These French real-world data confirm the effectiveness, safety, and tolerability of B/F/TAF in TN and TE participants with a high prevalence of comorbidities.

## Highlights

• Bictegravir/emtricitabine/tenofovir alafenamide (B/F/TAF) confirmed a high effectiveness of 92% in persons with HIV after 12 months.
• No major mutations associated with resistance to B/F/TAF emerged.
• Persistence to B/F/TAF was high, with no discontinuation for virologic reasons.
• B/F/TAF was well tolerated, with no renal adverse reactions.
• Self-reported treatment satisfaction increased in pre-treated individuals.

## Introduction

Because HIV antiretroviral therapy (ART) is lifelong, treatment must be effective, tolerable, safe and convenient to use. The bictegravir (B)/emtricitabine (F)/tenofovir alafenamide (TAF) (B/F/TAF) single-tablet regimen (a co-formulation of the integrase strand transfer inhibitor [INSTI] B, and the nucleo[s/t]ide reverse transcriptase inhibitors [NRTIs] F, and TAF) is among the recommended regimens for first-line ART [1].

Several phase III randomized trials have demonstrated the non-inferiority, safety, and tolerability of B/F/TAF without the development of resistance-associated mutations (RAMs) compared with INSTI-based treatment with dolutegravir in treatment-naive (TN) individuals with HIV [2,3] and treatment-experienced (TE) individuals switching from boosted protease inhibitor (PI)–based regimens [4,5]. These findings were consistent with *in vitro* studies suggesting a higher resistance barrier of the second-generation INSTI B than the first-generation INSTIs raltegravir and elvitegravir [6]. B/F/TAF was approved by the European Medicines Agency on June 21, 2018 for use in TN and TE people living with HIV (PWH) without current or past evidence of viral resistance to the INSTI class, emtricitabine, or tenofovir. Recently published 5-year outcomes confirm the favorable safety profile and high resistance barrier of B/F/TAF in the longer term [[7], [8], [9], [10], [11]].

The aim of the BICSTaR (Bictegravir Single Tablet Regimen) cohort study is to evaluate the effectiveness, safety, and self-reported quality of life in PWH receiving B/F/TAF in routine clinical care. Of the 2379 PWH recruited worldwide, 2142 were prospectively enrolled. Multinational data from 2074 individuals have been published [12].

We present the 12-month results from the French subcohort to provide granular insights at country level.

## Patients and Methods

### Study setting and study population

BICSTaR is a 2-year (extended to 5 years in France, Canada, and Germany), multinational, observational, non-interventional cohort study of adult TN and TE participants with HIV receiving B/F/TAF (50/200/25 mg) after June 2018. Data were collected in five cohorts representing 14 countries: (i) France, Germany, Ireland, Italy, the Netherlands, Spain, Turkey, and the United Kingdom; (ii) Canada; (iii) Israel; (iv) Singapore, South Korea, and Taiwan; and (v) Japan. In France, data collection was prospective and in accordance with the European Union label.

Participants could be enrolled in this study after the physician had independently decided to treat with B/F/TAF in accordance with the approved indication label in Europe and the written informed consent of the participants.

This 12-month analysis included all participants in the French subcohort of the European BICSTaR study with at least one baseline (BL) and one month 12 (M12) visit (within a time window of 547 days after treatment initiation), as well as participants who had discontinued B/F/TAF before the data cut-off date of February 18, 2022 (full analysis set).

### Primary and secondary end points

The primary end point was virologic response, defined as plasma HIV-1 RNA <50 copies/mL, at 12 months after initiation of or switch to B/F/TAF.

Secondary end points included HIV-1 RNA suppression (<50 copies/ml) at months 3 and 6, the development of resistance after enrollment, and changes in clusters of differentiation (CD) 4 cell count and CD4/CD8 ratio at M12. Additional secondary end points include cumulative incidence rates of drug-related adverse events (DRAEs) and drug-related serious adverse events (DRSAEs), as well as changes in body weight and body mass index (BMI).

Additional exploratory end points include reasons for ART initiation in TN and for switching to B/F/TAF in TE participants, persistence on B/F/TAF, and reasons for any discontinuation, as well as the patient-reported outcomes of symptom burden and treatment satisfaction.

### Statistical methods and outcome measures

#### Statistical analyses

All analyses were conducted for the full population and stratified by TN or TE participants. Further stratifications were performed for sex at birth and age. Descriptive analyses were performed on observed data using the SAS 9.4 software package (SAS Institute Inc., Cary, NC, USA). There was no hypothesis testing; statistical tests were applied only for exploratory analyses, provided that n ≥20, without adjustment for multiple testing.

#### Outcome measures

HIV-1 RNA suppression at M12 was defined as plasma HIV-1 RNA <50 cp/mL, using two approaches: a missing = excluded (M = E) approach (only HIV-1 RNA data collected within the 12-month window while on study treatment were analyzed) and a discontinuation = failure (D = F) approach (with imputation for participants who discontinued B/F/TAF before the M12 time window).

Safety parameters included the cumulative incidence of reported DRAEs or DRSAEs, as well as changes in body weight and laboratory parameters. All medical events were coded using the Medical Dictionary for Regulatory Activities system organ classes and preferred terms.

Standardized questionnaires were used to assess symptom burden and treatment satisfaction. The HIV Symptom Index (HIV-SI) questionnaire considers 20 symptoms associated with HIV infection [13]. The total score (i.e. the number of bothersome symptoms) ranges from 0 to 20, with higher scores indicating more (bothersome) symptoms.

The HIV treatment satisfaction questionnaire encompasses a status (HIVTSQs) and a change (HIVTSQc) version with 10 items each [14,15]. HIVTSQs scores range from 0 to 60, with higher scores indicating greater treatment satisfaction. During study follow-up, HIVTSQc scores range from −30 to +30, with positive scores indicating increased satisfaction.

## Results

### Study population/baseline characteristics

The French BICSTaR cohort enrolled 248 participants from 24 study sites. The full analysis set included 240 participants initiated on B/F/TAF between January and July 2019 for whom the inclusion criteria were confirmed, and one or more follow-up visits were documented (56 TN, 184 TE participants).

The study cohort was 79% male; one-third of TN (32%) and 59% of TE participants were at least 50 years of age. BL characteristics are shown in Table 1.

**Table 1:** Baseline characteristics and comorbidities/concomitant medications.

Demographics	All (n = 240)	TN (n = 56)	TE (n = 184)
Male sex, n (%)	190 (79)	51 (91)	139 (76)
Age, years, median (Q1, Q3)	50 (40, 58)	44 (34, 54)	52 (43, 58)
Age ≥50 years, n (%)	126 (53)	18 (32)	108 (59)
Weight, kg, median (Q1, Q3)	72 (64, 82)^a^	70 (63, 78)^b^	73 (64, 85)^c^
Body mass index, kg/m^2^, median (Q1, Q3)	24 (22, 26)^d^	22 (21, 26)^e^	24 (22, 26)^f^
Race, n (%) White	166 (69)	38 (68)	128 (70)
Black	45 (19)	10 (18)	35 (19)
**HIV-related characteristics**	**All (n = 240)**	**TN (n = 56)**	**TE (n = 184)**
Number of previous antiretroviral therapy regimens, median (Q1,Q3)		—	3 (2, 6)
Time from HIV diagnosis to bictegravir/emtricitabine/tenofovir alafenamide start, median (Q1,Q3)		19 days (9, 42)^b^	—
HIV-1 RNA, log_10_ cp/ml, median (Q1,Q3)	1.4 (1.3, 2.9)^g^	5.0 (4.1, 5.4)^h^	1.3 (1.3, 1.6)^i^
HIV-1 RNA >100,000 cp/ml, n (%)	25 (11)^g^	24 (44)^h^	1 (0.6)^i^
HIV-1 RNA <50 cp/ml, n (%)	154 (70)^g^	—	154 (93)^i^
CD4 count, cells/µl, median (Q1,Q3)	533 (350, 830)^j^	341 (132, 490)^k^	659 (419, 928)^l^
CD4 count, <200 cells/µl, n (%)	22 (11)^j^	17 (32)^k^	5 (3)^l^
CD4/CD8 ratio, median (Q1,Q3)	0.8 (0.4, 1.2)^m^	0.3 (0.2, 0.7)^n^	1.0 (0.6, 1.3)^o^
CDC Stage C (AIDS), n (%)	52 (22)^p^	8 (15)^k^	44 (24)^q^
History of virological failure, n (%)		—	43 (24)^r^
**Comorbidities/concomitant medication, n (%)**	**All (n = 240)**	**TN (n = 54)**	**TE (n = 184)**
Any comorbidity	151 (63)	26 (48)	125 (68)
None	87 (37)	28 (52)	59 (32)
1-2	96 (40)	19 (35)	77 (42)
≥3	55 (23)	7 (13)	48 (26)
Category^s^ (in ≥10% participants)			
Neuropsychiatric disorder	44 (18)	6 (11)	38 (21)
Hyperlipidemia	35(15)	2 (4)	33 (19)
Hypertension	32 (13)	7 (13)	25 (14)
Osteopathic disorder	23 (10)	1 (2)	22 (12)
Any concomitant medication, n (%)	129 (57)^t^	27 (50)^k^	102 (59)^u^

CD, clusters of differentiations; Q, quartile;

a n = 218

b n = 53

c n = 165

d n = 213

e n = 51

f n = 162

g n = 221

h n = 55

i n = 166

j n = 209

k n = 54

l n = 155

m n = 189

n n = 48

o n = 141

p n = 234

q n = 180

r n = 176

s Participants may be counted more than once

t n = 228

u n = 174.

#### Comorbidities and concomitant non-ART medications

At BL, comorbidities or co-infections were reported for 63% (n = 151 of 240) of participants. Most common comorbidities were neuropsychiatric disorders, followed by hyperlipidemia and hypertension (Table 1). Eight TE (4%) and no TN experienced chronic hepatitis B; three TE (2%) and one TN (2%) had chronic hepatitis C.

More than half of the participants (57%, n = 129 of 228) were taking at least one concomitant non-ART medication at the time of B/F/TAF initiation. Among participants <50 years of age, this percentage was 45% (n = 49 of 109), whereas among participants ≥50 years old (n = 80 of 119), it was 67%. Moreover, this age group was taking a higher quantity with 21% (n = 21 of 102) (vs 4% [n = 1 of 27] of those aged <50 years) taking at least five concomitant medications (defined as single active ingredients as by World Health Organization generic names). Looking at the classes of concomitant medications and comparing their use between age groups <50 and ≥50 years, analgesics were used by 4% of the TE participants aged <50 years compared with 17% in the group aged ≥50 years, and 2% of the younger participants received lipid-modifying agents compared with 16% of the group aged ≥50 years. Similarly, renin-angiotensin system agents, antithrombotics, and β blockers were used by 1% of the younger group compared with 16%, 13%, and 11%, respectively, of the older participants. Psycholeptics in various forms were prescribed to 4% of the participants <50 years of age versus 10% of those ≥50 years of age.

#### Antiretroviral treatment history and reason for B/F/TAF initiation

TE participants had received a median of 3 (interquartile range [IQR]: 2-6) previous ART regimens (20% received one, 32% had received two or three, and 48% had received at least four regimens). Among the 184 TE participants, 60% had been previously exposed to INSTIs, 68% to PIs, 52% to non-NRTIs, and 98% to NRTIs; 78% had received a regimen containing tenofovir disoproxil fumarate (TDF). A history of virologic failure was identified in 24% (n = 43 of 176) of the TE participants.

**Figure 1:**
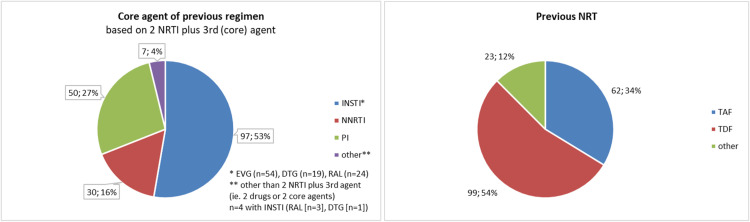
Previous antiretroviral therapy taken just before bictegravir/emtricitabine/TAF.

DTG, dolutegravir; EVG, elvitegravir; INSTI, integrase strand transfer inhibitor; NRTI, nucleos(t)ide reverse transcriptase inhibitor; NNRTI, non-NRTI; PI, protease inhibitor; RAL, raltegravir; TAF, tenofovir alafenamide; TDF, tenofovir disoproxil fumarate.

Before initiation of B/F/TAF, 62 TE (34%) were on F/TAF-containing ART, of whom 84% were on elvitegravir/cobicistat/tenofovir alafenamide/emtricitabine and the remaining 16% were on combinations with boosted darunavir, raltegravir, or rilpivirine (Figure 1).

Simplification of ART was the most common reason for switching to B/F/TAF for the majority (54%) of TE participants, followed by side effects of the previous regimen (28%). In the TN group, 93% started B/F/TAF as early treatment according to guidelines.

### Virologic outcomes

**Figure 2:**
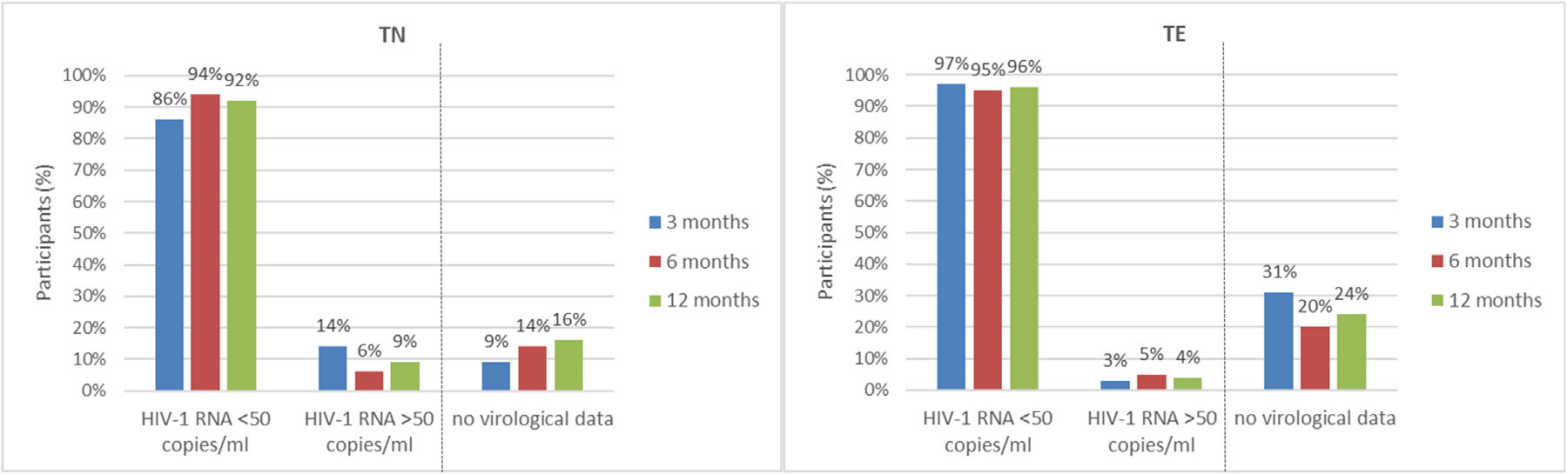
Effectiveness at 3, 6, and 12 months (missing = excluded analysis) in TN participants and TE participants. N = 56; month 12: HIV-1 RNA data for n = 47 and no data for n = 9 (16% of TN) N = 184; month 12: HIV-1 RNA data for n = 139 and no data for n = 45 (24% of TE). TE, treatment-experienced; TN, treatment-naïve.

At 12 months, HIV-1 RNA was <50 cp/ml in 95% (n = 177 of 186) of the participants (92% of TN [n = 43 of 47] and 96% of TE [134 of 139]) in the M = E analysis. HIV-1 RNA <50 cp/ml at 3, 6, and 12 months is shown in Figure 2. Furthermore, HIV-1 RNA was <50 cp/ml in 95% (n = 36/38) of women (female at birth) and 95% (n = 98/103) of those ≥50 years of age.

Results were similar in the D = F analysis (overall 92% [n = 177 of 193], no viral load data n = 47; TN: 92% [n = 43 of 47], no viral load data for n = 9; TE: 92% [n = 134 of 146], no viral load data for n = 38). The lack of virologic data (in 20% of the full analysis set) was either due to missing HIV-1 RNA data (n = 33, 14%) or study withdrawal/discontinuation (n = 14, 6%).

Eight TN participants had plasma HIV-1 RNA levels ≥50 copies/ml at M12 with 321, 297, 229, 106, 80, 72, 53, and 52 cp/ml. Three of them had started B/F/TAF with BL levels >500,000 cp/ml, of which two with >1.5 million cp/ml had not reached undetectability by M12. For four of these eight TN, undetectable HIV-1 RNA levels were measured beforehand, the M12 value possibly indicating a viral blip.

Of the 12 TE participants who were viremic at BL, nine of 10 (90%) had <50 copies/ml at M12. Only one had a plasma HIV-1 RNA >200 cp/ml (297 cp/ml), with no BL resistance documented to B/F/TAF. Two of 12 were lost to follow-up: one with a plasma HIV-1 RNA of 53 cp/ml at month 3 and 1 with no data beyond BL.

### Immunologic outcomes

The median absolute CD4 cell count in TNs changed from 341 cells/µl (IQR: 132-490) at BL (n = 54 of 56) to 560 cells/µl (IQR: 426-824) at M12 (n = 42 of 56), reflecting a median absolute CD4 change of +196 cells/μl (IQR: 121, 346; n = 40 of 56; *P* <0.001). The median CD4/CD8 ratio increased by 0.29 (n = 36 of 56; *P* <0.001) from 0.32 (IQR: 0.17-0.66) at BL (n = 48 of 56) to 0.74 (IQR: 0.36-1.05) at M12 (n = 39 of 56). Approximately one-third of TN participants (31%) had a ratio of 0.9 or higher at M12 (n = 12 of 39) compared with 13% (n = 6 of 48) at BL.

The median CD4 cell count remained stable in TE participants (BL: 659/µl [IQR: 419-928], n = 155 of 184; M12: 689/µl [IQR: 472-857], n = 131 of 184). The median change was +13/μl (IQR: −81 to +83; n = 116 of 184; *P* = 0.307). There was no significant change in median CD4/CD8 ratio (BL: 0.93 [IQR: 0.58-1.30], n = 141 of 184); M12: 0.90 [IQR: 0.67-1.28], n = 118 of 184) (*P* = 0.117). At M12, 54% of TE participants (n = 64 of 118) had a ratio of ≥0.9 compared with 53% (n = 74 of 141) at BL.

### Resistance analysis outcomes

Preexisting genotypic resistance mutations were identified at BL in 54% of participants (29 of 54 TN, 98 of 183 TE). Furthermore, two of 54 (4%) of TN and 42 of 180 (23%) of TE had at least one major resistance-associated mutation. In the TN group, there was one NRTI RAM (M41L) and one PI RAM (D30N), whereas in the TE group, at least one major mutation associated with any of the drug classes was present: non-NRTI (14%), PI (5%), NRTI (14%), and INSTI (0.6%, T97A).

A total of 20 participants (18% of those with resistance data available for major mutations, n = 112), all in the TE group, had the M184V/I mutation and one (0.9%) had a T97A mutation. All but one of these participants (one with M184V/l) had HIV-1 RNA <50 cp/mL at BL. In these 21 participants with the preexisting M184V/I or T97A mutations, the last available HIV-1 RNA was <50 cp/ml (n = 19 at M12, n = 1 [with M184V/I] at month 6, and n = 1 [with M184V/I] had only BL data available).

Five resistance assays were performed during follow-up; no emergence of major resistance mutations (no NRTI, three minor mutations to INSTI [two 74I and P145S/P]) to the components of B/F/TAF was reported. There were no discontinuations of B/F/TAF for virologic reasons.

### Treatment persistence and discontinuation

Treatment persistence was high through M12, with only 6.7% (16 of 240) of participants discontinuing B/F/TAF within 12 months of initiation of treatment. The reasons for discontinuation were DRAEs 4.2% (10 of 240), investigator’s discretion 1.3% (three of 240), participant’s decision 0.9% (two of 240), and new treatment available 0.4% (one of 240). Of note, all participants who discontinued B/F/TAF (n = 16) had a last available HIV-1 RNA level of <50 cp/mL at the time of discontinuation.

### Safety

A total of 43 DRAEs were documented in six TN (11%) and 24 TE (13%) participants (Table 2).

**Table 2:** DRAEs up to month 12; by Medical Dictionary for Regulatory Activities coding.

DRAE, system organic class: preferred term (n >1)	Participants (N = 240)	Events
**Gastrointestinal disorders**: nausea (n = 4), diarrhea (n = 2), dyspepsia, flatulence (n = 2), abdominal pain, constipation	9 (3.8%)	11
**Nervous system disorders**: paresthesia (n = 3), disturbance in attention, dizziness, headache, hypersomnia, hypokinesia, somnolence (n = 2)	6 (2.5%)	10
**Investigations**: weight increased (n = 8)	8 (3.3%)	8
**General disorders and administration site conditions**: asthenia (n = 3), fatigue	3 (1.3%)	4
**Psychiatric disorders**: depression, insomnia, sleep disorder (n = 2)	4 (1.7%)	4
**Musculoskeletal and connective tissue disorders**: arthritis, muscle spasms	2 (0.8%)	2
**Ear and labyrinth disorders**: vertigo	1 (0.4%)	1
**Hepatobiliary disorders**: hepatocellular injury	1 (0.4%)	1
**Skin and subcutaneous tissue disorders**: night sweats	1 (0.4%)	1
**Vascular disorders**: peripheral coldness	1 (0.4%)	1

DRAEs, drug-related adverse events.

The median time from B/F/TAF initiation to the first DRAE was 26 days (IQR: 13-95) for TN and 17 days (IQR: 1-189) for TE participants. There were no DRSAEs within the first 12 months.

**Figure 3:**
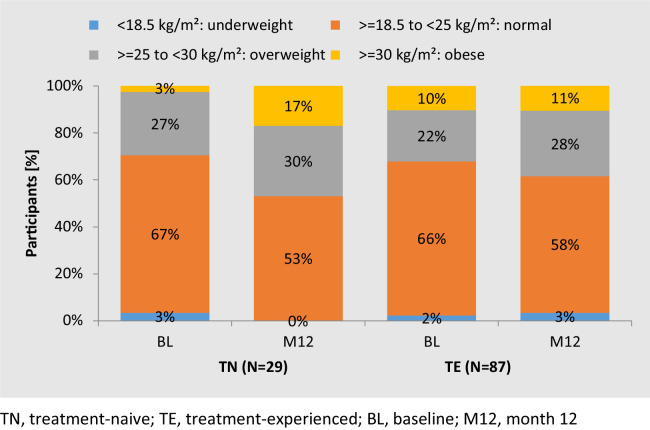
Body mass index distribution at BL and its change over the course of M12 in TN and TE with data available at both time points.

Weight and BMI were analyzed for participants with available data at BL and M12 (TN: n = 30 of 56; TE: n = 93 of 184). The median BL body weight was 69 kg (IQR: 63-78) for TN and 71 kg (IQR: 64-83) for TE participants. The median BL BMI was 22 kg/m^2^ (IQR: 21-26) for TN and 24 kg/m^2^ (IQR: 22-27) for TE participants. The median change in weight and BMI from BL to M12 was +6.5 kg (IQR: 3.0-8.0) and +2.2 kg/m^2^ (IQR: 1.0-2.9), respectively, for TN participants (*P* <0.001); it was +1.0 kg (IQR: −0.5-+3.0) and 0.4 kg/m^2^ (IQR: −0.2 to +1.0), respectively, for TE participants (*P* = 0.003). BMI distribution and its changes are shown in Figure 3.

Further stratification of median weight in TN by age revealed a greater weight increase in those ≥50 years old (BL: 68.2 kg, n = 17; 12M: 81.5 kg, n = 11) than those <50 years (BL: 70.0 kg n = 36; 12M: 72.2 kg, n = 22, *P* <0.001). In TE, on the other hand, those ≥50 years of age experienced a decrease (BL 73.5 kg, n = 100; 12M: 71.0 kg, n = 67, *P* <0.001), which was driven by the subset without previous TDF exposure. In women (female at birth), the median weight changed from 55.0 to 74.5 kg in TN (n = 4); in TE, it remained at 67.0 kg (n = 25), balanced by a weight increase with and decrease without previous TDF use (data not shown).

Two-thirds of TN (67%, n = 20 of 30) gained more than 5% body weight, whereas 13 of 30 (43%) gained more than 10%. None of these individuals had discontinued treatment at M12.

Weight gain of >5% was observed in 18 of 93 TE participants (19%), whereas five TE (5%) gained more than 10% (PWH with weight gain >10% are described in the Supplementary Table). Three TE participants decided to discontinue treatment with B/F/TAF because of weight gain.

Of note, 99 (54%) of all TE had taken a TDF-based regimen immediately before B/F/TAF. TE with immediate previous TDF exposure experienced a 2.0-kg (IQR: 0-3.0) and 0.7-kg/m^2^ (IQR: 0-1.1) increase in median weight and BMI, respectively (*P* = 0.003), compared with 0.5 kg (IQR: −1.0 to 2.9) and 0.1 kg/m^2^ (IQR: −0.5 to 0.9) in TE participants without exposure (*P* = 0.268).

At BL, the median estimated glomerular filtration rate for participants with BL and M12 values was 91.3 ml/min/1.73 m^2^ (IQR: 85.2-111.6) for 22 of 56 TN and 86.8 ml/min/1.73 m^2^ (IQR: 73.1-111.5) for 71 of 184 TE participants with available data. The median change from BL to M12 was −7.2 ml/min/1.73 m^2^ (IQR: −11.8 to +4.7) for 22 of 56 TN with (*P* = 0.017), and −3.6 ml/min/1.73 m^2^ (IQR: −13.0 to +4.9) for 71/184 TE participants (*P* = 0.008). There were no discontinuations due to renal adverse events related to B/F/TAF during follow-up.

No significant changes were observed from BL to M12 in cholesterol, low-density lipoprotein, high-density lipoprotein, total cholesterol / high-density lipoprotein ratio, and triglycerides (Supplementary Figure).

### Patient-reported outcomes

A total of 37 TN (66%) and 104 TE (57%) participants completed the HIV-SI questionnaire at BL and at M12. At BL, the median symptom distress score was 6.0 (IQR: 3.0-8.0) for TN and 5.0 (IQR: 1.0-8.0) for TE participants.

At M12, the median change in bothersome symptoms was −3.0 (IQR: −6.0 to 0.0) (*P* = 0.005) for TN and 0.0 (IQR: −2.0 to +2.0) (*P* = 0.391) for TE participants. Of note, women (female at birth) in the TE group experienced a reduction of −2.0 (IQR: −4.5 to 0) (*P* = 0.077).

Symptom distress in TE was further analyzed according to a history of or current neuropsychiatric symptoms at BL (n = 38, 21% of TE). The HIV-SI questionnaire was completed at BL and M12 by 22 of 38 TE participants with and 82 of 146 TE without a neuropsychiatric disorder; the median BL scores for bothersome symptoms were 8.0 (IQR: 5.0-11.0) and 3.0 (IQR: 1.0-7.0), respectively.

The median changes at M12 were −2.0 (IQR: −5.0 to 0.0) (*P* = 0.01) and 0.0 (IQR: −2.0 to +2.0) (*P* = 0.716), respectively (*P* = 0.002). Among other changes, the largest improvement in participants with neuropsychiatric disorders was seen in sleep disorders (−36% points).

At BL, 177 of 184 TE participants responded to the HIVTSQ. The median total score was 53.0 (IQR: 47.0-58.0). At M12, treatment satisfaction had increased significantly as reflected by a median HIVTSQ change score of 24.0 points (IQR: 12.0-29.0; *P* <0.001) (in 107 of 184 TE). The score in the subset women (female at birth) was 26.0 points (IQR: 19.0-29.0; n = 19).

## Discussion

The French BICSTaR subcohort study confirmed the favorable outcomes of B/F/TAF treatment observed in clinical trials with respect to the primary end point of virologic suppression, with 95% of the participants achieving or maintaining an HIV-1 RNA level <50 cp/ml after 12 months under treatment [2,4,5]. This is in concordance with real-world cohort data, as assessed in a meta-analysis by Chivite et al*.* [16]. There were no discontinuations due to lack of effectiveness. The virologic and immunologic outcomes and the safety profile seen in this French subcohort are similar to those reported in the multinational BICSTaR cohort [17]. Albeit, the weight gain in French TN was significantly higher. However, the French subcohort has unique characteristics: the population is older, with an approximately 10% higher proportion of participants over the age of 50 years, there are more females in the TE group, 5-10% more black individuals, and 5% more presented with a CD4 cell count of less than 200 cells/µl. Another difference lies in the ART history before the initiation of B/F/TAF. The French subcohort was more heavily pretreated with PIs and less with INSTIs. In addition, although half of the multinational cohort had received TAF (vs one-third TDF), the reverse was true in the French subcohort.

Of the TE participants, the vast majority were on suppressive ART before switching. One-quarter had experienced virologic failure in the past and one-quarter had a history of AIDS. Simplification of treatment and side effects were the most common reasons for switching to B/F/TAF.

Nearly one-half of TN participants started B/F/TAF with HIV-1 RNA >100,000 cp/ml. Immune reconstitution was favorable, with one-third achieving normalization of the CD4/CD8 ratio at 12 months.

These results, together with the good tolerability of the single-tablet regimen, explain the high persistence. Discontinuation was rare. Consequently, there were only five cases of resistance testing in this cohort during follow-up, with no major RAMs to the components of B/F/TAF. Moreover, in the 21 participants who had evidence of preexisting RAMs (M184V/I or T97A) at BL, the last available HIV-1 RNA was undetectable.

The evaluation of safety parameters in this cohort showed a slightly lower estimated glomerular filtration rate, consistent with the inhibitory effect of B on renal tubular creatinine secretion, resulting in an increase in serum creatinine without affecting glomerular filtration [1]. There were no discontinuations due to renal adverse events associated with B/F/TAF. Other known drug-related adverse events of B/F/TAF, such as headache or sleep disorders [18], were uncommon in our cohort; DRAEs accounted for only 4% of discontinuations in 12 months. Hoffmann et al. had observed that neuropsychiatric adverse events (AEs) were more likely to lead to discontinuation of dolutegravir in a real-world setting than in clinical trials. They further found that B/F/TAF discontinuation rates due to neuropsychiatric AEs were similar to those of dolutegravir-containing regimens at least in the short-term of 6 months (3%) and, thus, higher than in clinical trials [19]. In our cohort, the corresponding 1-year discontinuation rate was 3%, whereas in those with a neuropsychiatric disorder at BL, an improvement has been seen (although not statistically significant).

Patient-reported outcomes were not available for all participants in our analysis. However, the results were consistent with those of previous studies [20] where PWH initiating B/F/TAF reported a decrease in bothersome symptoms such as depression, nausea, or sleep disorders. In a small subgroup of TE participants with neuropsychiatric symptoms in BICSTaR, the overall symptom burden at BL was higher than in other participants. Within 12 months, we observed a significant reduction in symptom burden in this subgroup, with improvements in symptoms associated with neuropsychiatric disorders. Of note, week 48 results of the Ebony study [21] showed significant improvement in sleep, memory/attention problems, and/or psychiatric symptoms after switching to B/F/TAF. Satisfaction with treatment increased in those who switched to B/F/TAF, with a change score of +20.

There has been concern about patterns of weight change with contemporary antivirals, as noted in recent guidelines [1]. Weight gain has been observed with INSTI use, particularly, in combination with TAF, but is known to be multifactorial with demographic and HIV-related factors aside those stemming from ART use [1,22,23]. A large retrospective study of more than 2200 participants in the US Trio Health HIV database [24] found no difference in weight gain between second- and first-line INSTIs but did find greater weight gain in participants switching from TDF- to TAF-containing regimens. Individuals switching from efavirenz or TDF experienced the greatest weight gain in a pooled analysis by Erlandson et al., with 6% gaining more than 10%; moderate increases were common but leveled off after 48 weeks of observation [25]. Since TDF and efavirenz are known to have weight-suppressive effects [1], which are reversed after discontinuation or switching, it is important to note that these effects may not be seen in all patients and that in our study population, more than half were on TDF before switching. In the French DAT´AIDS cohort, Hocqueloux et al. [26] showed a small but significant weight gain of +1.3 kg 12 months after switching from TDF- to TAF-based ART without a change in third agent, after adjusting for possible confounders such as sex, ethnicity, or age. Although weight gain was observed in the French BICSTaR cohort, the small number of participants with reported weight changes is a limitation. Several other confounding factors need to be considered, such as the “return to health effects” in TN participants, one-third of whom had advanced disease at enrollment with less than 200 CD4 cells/µl (and among whom we saw some outliers but no discontinuations due to body weight gain). On the other hand, in TN patients, an international cohort study highlights greater weight gain with TAF and/or INSTIs, with variations depending on sex, sexual orientation, and geographic origin [27]. Given the number of additional factors that potentially affect weight, studies comparing weight gain in PWH with that in the general population are needed to better understand potential differences and underlying mechanisms.

The absence of virologic data in 20% of participants may be regarded as a limitation; however, this reflects viral load monitoring in real-life settings as well as discontinuations before M12.

## Conclusion

The 12-month results of the prospective BICSTaR cohort, based on 240 TN and TE participants from France, confirm the high virologic effectiveness, safety, and tolerability of B/F/TAF use, with high persistence in a real-world setting. BICSTaR will continue for 24 months, with an additional 36-month extension in France.

## Declarations of Competing Interest

LH reports nonfinancial support from Gilead, Merck Sharp & Dohme, and ViiV Healthcare; honoraria payments and travel support for advisory board participation from Gilead, Merck Sharp & Dohme, and ViiV Healthcare; and personal consulting fees from Gilead, Merck Sharp & Dohme, and ViiV Healthcare, all outside the submitted work. FB reports nonfinancial support from Gilead, Merck Sharp & Dohme, and ViiV Healthcare; honoraria payments and travel support for advisory board participation from Gilead, and ViiV Healthcare; and personal consulting fees from Gilead, Merck Sharp & Dohme, and ViiV Healthcare, all outside the submitted work. CD declares fees and travel grants from Gilead, ViiV Healthcare and Merck. MV declares no conflict of interest. FT declares no conflict of interest. PL declares fees and travel grants from: Gilead, ViiV Healthcare and Merck. MK declares no conflict of interest. HC declares to receive grants to attend HIV and infectious diseases conferences from Gilead, ViiV and MSD. CDC declares fees and travel grants from Gilead, ViiV Healthcare and Merck. ACG declares no conflict of interest. OR declares personal consulting fees and honoraria payment from ViV Healthcare, Merck, Gilead, Pfizer and Moderna. DT, TC, FD, and SS are Gilead Sciences employees.

## Appendix Supplementary materials

Image, application 1

Image, application 2
