# Supplementary material for: Real-world effectiveness, safety, and health-related quality of life in people living with HIV receiving bictegravir/emtricitabine/tenofovir alafenamide—12-month results of the BICSTaR French cohort
Source: IJID Reg. 2025 Jun 17;16:100685. doi: 10.1016/j.ijregi.2025.100685 (PMC12270808; doi:10.1016/j.ijregi.2025.100685)
Supplement: Supplementary file 1 [file mmc1.docx]

### Figure - Lipids at baseline and the change at months 12 in TN and TE with data available at both time points

HDL, high-density lipoproteins; LDL, low-density lipoproteins; IQR, interquartile range; TC, total cholesterol; TE, treatment-experienced; TN, treatment-naïve
