# Supplementary material for: Real-world effectiveness, safety, and health-related quality of life in people living with HIV receiving bictegravir/emtricitabine/tenofovir alafenamide—12-month results of the BICSTaR French cohort
Source: IJID Reg. 2025 Jun 17;16:100685. doi: 10.1016/j.ijregi.2025.100685 (PMC12270808; doi:10.1016/j.ijregi.2025.100685)
Supplement: Supplementary file 2 [file mmc2.docx]

### Supplemental Table - Participants with weight gain >10% (n=18)

| **Study arm** | **Gender** | **Age at BL** | **CD4 count [cells/µL] at BL** | **HIV-1 RNA [log cp/mL] at BL** | **ART at BL** | **Weight at BL** | **Weight at M12** | **BMI at BL** | **BMI at M12** | **Weight increase [%]** | **DRAE** | **Disc.** | **Medical conditions documented at BL** |
| --- | --- | --- | --- | --- | --- | --- | --- | --- | --- | --- | --- | --- | --- |
| TN | m | 19 | 834 | 4.00 | n/a | 56 | 64 | 19.8 | 22.7 | 14.3 | no | no | neuropsychiatric disorder |
| TN | m | 27 | 377 | 3.51 | n/a | 64 | 72 | 19.1 | 21.5 | 12.5 | no | no |  |
| TN | m | 38 | 440 | 4.03 | n/a | 89 | 100 | 27.8 | 31.2 | 12.4 | no | no | hypertension, sleep apnoea |
| TN | m | 38 | 151 | 6.2 | n/a | 65 | 98 | 21.7 | 32.7 | 50.8 | no | no | neuropsychiatric disorder, herpes zoster, pneumonia bacterial, syphilis, thrombocytopenia |
| TN | m | 42 | 1 | 5.26 | n/a | 63 | 72 | 20.8 | 23.8 | 14.8 | no | no |  |
| TN | m | 44 | 436 | 3.51 | n/a | 76 | 84 | 25.7 | 28.4 | 10.5 | yes | no | visual acuity reduced |
| TN | m | 45 | 20 | 5.96 | n/a | 58 | 66 | 21.6 | 24.5 | 13.8 | no | no |  |
| TN | f | 45 | 64 | 6.25 | n/a | 43 | 75 | 16.8 | 29.3 | 74.4 | no | no |  |
| TN | m | 51 | 89 | 5.18 | n/a | 72 | 82 | 22.0 | 25.0 | 13.5 | no | no |  |
| TN | m | 56 | 59 | 5.39 | n/a | 68 | 96 | 19.9 | 28.0 | 40.8 | no | no |  |
| TN | m | 58 | 120 | 6.74 | n/a | 68 | 75 | 23.5 | 26.0 | 10.3 | no | no | cardiovascular |
| TN | f | 64 | 474 | 4.41 | n/a | 64 | 77 | 28.4 | 34.2 | 20.3 | no | no | neuropsychiatric disorder, lumbar hernia |
| TN | m | 71 | 10 | 4.99 | n/a | 60 | 68 | 19.2 | 21.7 | 13.3 | no | no |  |
| TE | m | 26 | - | 1.87 | RPV/TDF/ FTC | 67 | 74 | 21.9 | 24.2 | 10.4 | no | no | headache |
| TE | f | 43 | 248 | 1.30 | EVG/COB/ TAF/FTC | 62 | 69 | 23.9 | 26.6 | 11.3 | no | no |  |
| TE | m | 52 | 350 | 1.28 | DTG/TDF/ FTC | 62 | 72 | 21.7 | 25.2 | 16.1 | no | no | cardiovascular, hypertension, osteopathic disorder, myocardial ischaemia, renal insufficiency |
| TE | m | 54 | - | 1.41 | LPV/r/ TDF/FTC | 79 | 89 | 26.1 | 29.4 | 12.7 | no | no | asthma, cardiovascular, hyperlipidemia, hypertension, lateral medullary syndrome |
| TE | m | 63 | 408 | 1.28 | EVG/COB/  TAF/FTC | 79 | 88 | 24.4 | 27.2 | 11.4 | no | no | diabetes mellitus, COPD, neuropsychiatric disorder |

BL, baseline; TN, treatment-naive; TE, treatment-experienced; m, male; f, female; RPV/TDF/FTC, Rilpivirin/Tenofovir/Emtricitabin; EVG/COB/TAF/FTC, Elvitegravir/Cobicistat/Tenofovir/Emtricitabin; DTG, Dolutegravir; LPV/r, Lopinavir/Ritonavir; BMI, body mass index; DRAE, drug related adverse event; Disc., discontinuation; n/a, not applicable.
